# Right colic artery anatomy: a systematic review of cadaveric studies

**DOI:** 10.1007/s10151-017-1717-6

**Published:** 2017-12-02

**Authors:** M. Haywood, C. Molyneux, V. Mahadevan, N. Srinivasaiah

**Affiliations:** 1 Department of Anatomy and Physiology, Institute of Health Sciences Education, Barts and the London School of Medicine and Dentistry, Queen Mary University of London, London, E1 4NS UK; 2 Department of Anatomy, The Royal College of Surgeons of England, London, UK; 3 Department of Colorectal Surgery, The Princess Alexandra Hospital, Harlow, Essex UK

**Keywords:** Oncosurgery, Right colic artery, Complete mesocolic excision

## Abstract

**Background:**

Complete mesocolic excision for right-sided colon cancer may offer an oncologically superior excision compared to traditional right hemicolectomy through high vascular tie and adherence to embryonic planes during dissection, supported by preoperative scanning to accurately define the tumour lymphovascular supply and drainage. The authors support and recommend precision oncosurgery based on these principles, with an emphasis on the importance of understanding the vascular anatomy. However, the anatomical variability of the right colic artery (RCA) has resulted in significant discord in the literature regarding its precise arrangement.

**Methods:**

We systematically reviewed the literature on the incidence of the different origins of the RCA in cadaveric studies. An electronic search was conducted as per Preferred Reporting Items for Systematic Reviews and Meta-analyses recommendations up to October 2016 using the MESH terms ‘right colic artery’ and ‘anatomy’ (PROSPERO registration number CRD42016041578).

**Results:**

Ten studies involving 1073 cadavers were identified as suitable for analysis from 211 articles retrieved. The weighted mean incidence with which the right colic artery arose from other parent vessels was calculated at 36.8% for the superior mesenteric artery, 31.9% for the ileocolic artery, 27.7% for the root of the middle colic artery and 2.5% for the right branch of the middle colic artery. In 1.1% of individuals the RCA shared a trunk with the middle colic and ileocolic arteries. The weighted mean incidence of 2 RCAs was 7.0%, and in 8.9% of cadavers the RCA was absent.

**Conclusions:**

This anatomical information will add to the technical nuances of precision oncosurgery in right-sided colon resections.

## Introduction

Right hemicolectomy is the current operative standard for right-sided and transverse colon cancer [1, 2]. The ileocolic vessels are consistently ligated, but the same is not necessarily true of the right colic artery (RCA), a factor which the authors believe may lead to oncologically inferior outcomes [3, 4]. In the era of precision surgery, complete mesocolic excision (CME) is gathering favour as the procedure of choice for treating malignancy of the colon [5, 6]. The features of this technique include sharp dissection in the mesocolic plane, a high vascular tie of the feeding vessel (ensuring adequate horizontal length) and resection of the relevant lymphovascular package draining the tumour [7]. It is hoped that application of these principles will produce better oncological and functional outcomes for patients. There are some prospective data to suggest that recurrence rates and survival may be superior with CME when compared to more traditional approaches [8]; however, evidence from randomised controlled trials is currently lacking and concerns have been raised regarding CME-related morbidity [9].

Precision surgery demands a meticulous understanding of the relevant lymphovascular anatomy (obtained either pre- or intraoperatively) and a tailored dissection in each case. Considerable problems may be encountered when applying these principles to right-sided colon resections given the highly variable nature of the right colic feeding vessel [5, 6]. Indeed, there is significant disagreement in the anatomical literature regarding the incidence of its various origins [10–12]. Despite this problem CME is developing at a rapid rate, exemplified by the recent introduction of laparoscopic extended right hemicolectomy and robotic right hemicolectomy procedures employing CME principles [8, 9]. It is advancements such as these which have made it necessary to provide the surgical and anatomical communities with a contemporary and reliable overview of the anatomy of this area.

One of the largest reviews of the colonic blood supply was published in 1963, although no specific detail on the right colic vasculature was provided and many of the articles cited were in French or German [12]. A fairly comprehensive review of relevant cadaveric studies was published in 2016, but this was not truly systematic and the methodology was not fully disclosed [13]. One group did provide an overview of the vascular variation relevant to right colon cancer surgery; however, their focus was primarily on venous anatomy and the relationships of the different colic vessels rather than the origin of the right colic feeding vessel itself [14]. It was noted in 1996 that ‘even today, despite recent anatomical and surgical studies that widely emphasise the mistake, most medical textbooks still accept as a normal pattern of the colonic vasculature one that we believe is an infrequent variant’ [11]. Therefore, the current article attempts to address the following question: ‘What is the incidence of the different origins of the RCA as determined by a systematic review of observational human cadaveric studies?’ such that the RCA can be more clearly described in the modern era of CME.

## Materials and methods

### Search method

**Fig. 1:**
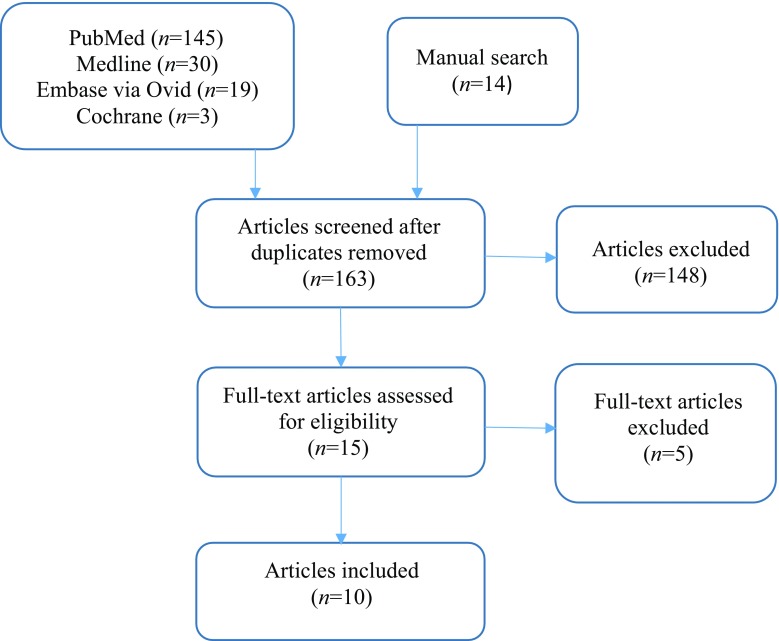
Right colic feeding vessel systematic review: preferred reporting items for systematic reviews and meta-analyses (PRISMA) flow diagram

This study was performed in accordance with Preferred Reporting Items for Systematic Reviews and Meta-analyses (PRISMA) recommendations (Fig. 1) [15]. An electronic search was conducted in October 2016 by 2 of the authors (MH and NS) in PubMed, Medline, Embase via Ovid and Cochrane from the inception of each database up to the search date. ‘Appendix’ details the strategies used. The search terms ‘right colic artery’ and ‘anatomy’ were applied; however, ‘cadaver’ was not included because we wished to maximise the scope of the initial literature trawl.

The search protocol was registered on PROSPERO (registration number CRD42016041578). MH and NS assessed all identified abstracts and titles of studies meeting the predetermined selection criteria to check eligibility (see ‘Inclusion and exclusion criteria’ section). Any discrepancies in data extraction were resolved by discussion between the authors. The bibliography of each suitable article was checked for additional references, particularly historical articles beyond the scope of the databases used.

### Inclusion and exclusion criteria

Observational human cadaveric articles examining the arrangement of the vasculature of the gastrointestinal tract with abstract and main text written in English were considered. Studies reporting radiological or intraoperative data were outside the scope of this review and were excluded, as were animal-based studies and review articles that did not contain original data.

### Outcome measures

The numbers of cadavers examined for each dissection series and the incidence of the right colic vessel taking origin from different parent vessels (i.e. superior mesenteric artery (SMA), ileocolic artery (ILC), right branch of the (MCA), root of the MCA or absent right colic vessel) were noted.

### Statistical analysis

The incidence with which the RCA arose from a given origin in each study was recorded in a Microsoft Excel spreadsheet and expressed as a percentage of the total number of RCAs identified in that study. This enabled cadavers possessing more than one RCA to be included in the analysis, with each RCA treated as a separate entity. The number of cadavers without an RCA and those possessing 2 or 3 RCAs were expressed as percentages of the total number of cadavers in each series. The weighted mean incidence of the RCA arising from each of the described origins was then calculated. Weighted mean incidence accounts for the sample size of each study; therefore, the larger the study the more influence it exerted over the mean in proportion to the value *n.*

## Results

The search yielded 197 citations (PubMed *n* = 145, Medline *n* = 30, Embase via Ovid *n* = 19, Cochrane *n* = 3) (Fig. 1). Fourteen additional papers were obtained through selected article reference lists to give a total of 211 articles. After removal of 48 duplicates, 163 titles and abstracts were screened and 148 excluded as they did not meet inclusion criteria. Of 15 full-text articles, five were excluded: one study grouped RCAs arising from the right branch of the middle colic artery (RBMC) and root of the MCA together, thus precluding calculation of an accurate incidence for each origin [16]; one study only considered a right colic vessel to be present if it originated directly from the superior mesenteric artery (SMA), thus giving a spuriously high incidence for the absence of an RCA [17]; one study focused on the marginal artery of Drummond and expressed the data using schematic drawings, precluding accurate interpretation of RCA arrangement [18]; one study did not account for each cadaver in its series and the RCA data were limited [19]; and one study used an unrecognised classification system of superior, middle and inferior RCAs, thus precluding the integration of its data into this review [20]. Ten studies were suitable for inclusion in the final analysis (Table 1).

**Table 1:** Incidence of different origins of the right colic artery

Author and year	Number of cadavers	Number of cadavers with absent right colic feeding vessel (%)	Number of RCAs	Incidence of RCA (i.e. SMA origin) (%)	Incidence of right colic branch (%)				Number of cadavers with 2 RCAs (%)	Number of cadavers with 3 RCAs (%)
					ILC	RBMC	Root of MCA	Trunk shared with MCA and ILC		
Haywood [21]	25	2 (8.0)	27	33.3	14.8	33.3	18.5	–	4 (16.0)	–
Gamo [22]	50	4 (8.0)	46	43.5	34.8	–	21.7	–	–	–
Acar [23]	12	1 (8.3)	11	45.6	54.5	–	–	–	–	–
Batra [24]	30	–	30	63.3	–	–	30.0	6.7	–	–
Jain [25]	20	–	20	75.0	25.0	–	–	–	–	–
Garcia-Ruiz [11]	56	–	56	10.7	66.0	–	23.3	–	–	–
Nelson [10]	50	1 (2.0)	57	29.8	26.3	–	43.9	–	8 (16.0)	–
Michels [12]	180	4 (2.0)	191	37.2	13.6	–	49.2	–	15 (8.3)	–
Sonneland [26]	600	74 (12.4)	581	35.5	38.0	–	24.8	1.7	47 (7.8)	4 (0.7)
Steward [27]	50	9 (18.0)	41	48.8	14.6	36.6	–	–	–	–
Weighted mean incidence (%)	1073 (total)	8.9	1060 (total)	36.8	31.9	2.5	27.7	1.1	7.0	0.4

*ILC* Ileocolic artery, *RBMC* right branch of the middle colic artery, *MCA* middle colic artery, *SMA* superior mesenteric artery, *RCA* right colic artery

In total, there were 1060 RCAs among 1073 cadavers across the ten selected studies. The weighted mean incidence of the RCA arising from the SMA was 36.8%, from the ILC 31.9%, from the root of the MCA 27.7%, from the RBMC 2.5% and from a trunk shared with the MCA and ILC 1.1%. The weighted mean incidence of an absent right colic vessel was 8.9%. The weighted mean incidence of a cadaver possessing two RCAs and three RCAs was 7.0 and 0.4%, respectively.

**Fig. 2:**
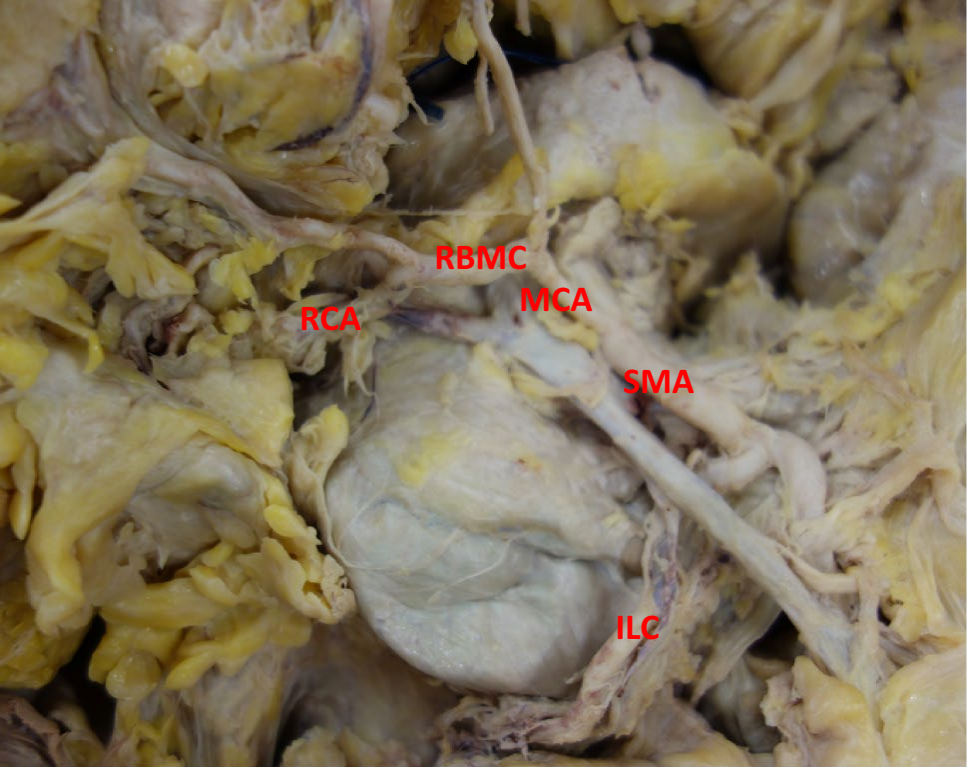
Right colic artery (RCA) arising from the right branch of the middle colic artery (RBMC). MCA middle colic artery; SMA superior mesenteric artery; ILC ileocolic artery (permission for reproduction granted by the Annals of the Royal College of Surgeons of England)

**Fig. 3:**
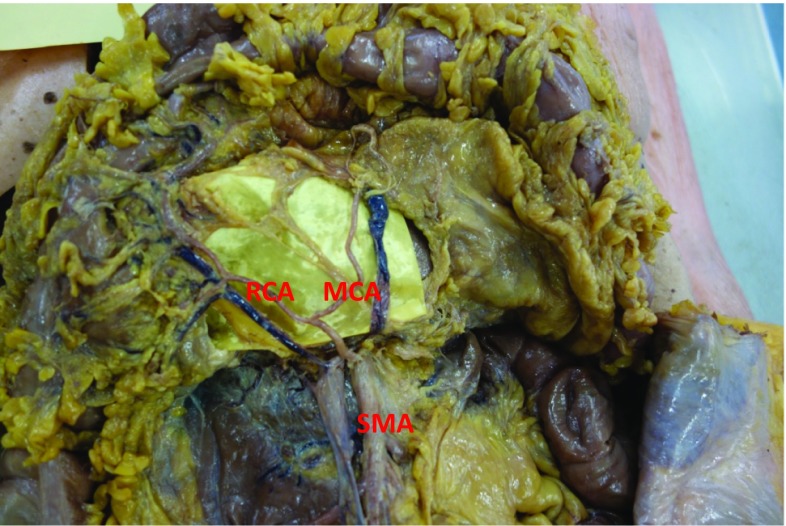
Right colic artery (RCA) arising from the root of the middle colic artery (MCA), i.e. before it bifurcates to give a left and right branch. SMA superior mesenteric artery (permission for reproduction granted by the Annals of the Royal College of Surgeons of England)

Figures 2 and 3 demonstrate the distinction between right colic vessels arising either from the root of the MCA (i.e. more proximally, before the MCA has bifurcated) or the RBMC (i.e. more distally, after the MCA has bifurcated).

## Discussion

Significant confusion has arisen over the decades as a result of the multiple attempts to provide a unifying definition for the right colic feeding vessel [21]. Traditionally within a surgical context it was regarded as any artery directed towards the hepatic flexure of the colon; however, no consideration was given to its origin [26, 27]. More recently some authors have begun to hone this classification by proposing that the term ‘right colic artery’ be reserved for a vessel arising independently from the SMA, whilst ‘right colic branch’ be applied in all other cases [11, 17]; this system is supported in the current review, owing to the high frequency with which the RCA arises from a vessel other than the SMA. Even though this is an improvement in the nomenclature, there is still considerable disparity surrounding the question of whether a right colic vessel is a branch of another larger artery or simply shares a trunk with it [11, 12, 26, 27]. The level of bifurcation and the presence of accessory right colic vessels further perpetuate the difficulty in defining this important vessel accurately. Whilst these quandaries may be regarded as academic by some, the authors argue that modern surgical techniques by their very nature are demanding an increasingly precise understanding of the relevant anatomy in order that more advanced operations may be performed effectively [13, 14, 22]. In the context of CME, by determining the exact origin of the right colic feeding vessel a high vascular tie and complete lymphovascular resection may be performed, resulting in an oncologically superior resection. A consistent method of describing the anatomy of the RCA will also aid in the generalisability and translation of future research into clinical practice. This notion of precise anatomical understanding is evident not only in CME for colon cancer, but also in fields as diverse as scaphoid reconstruction, hip arthroplasty, cochlear implantation and cervical cancer surgery [28–31].

We have shown the right colic feeding vessel origin to be highly variable, arising from the SMA, ILC, MCA root and RBMC in a decreasing order of frequency. It is absent in 8.9% of cases, and two RCAs may be found in 7.0% of individuals. Only a few smaller data series described the RBMC as a potential origin of the RCA [21, 27], resulting in a low (weighted) mean incidence for this particular arrangement. When all of this knowledge is applied to resection of a right colon tumour with CME technique, one is obligated to trace the tumour’s lymphovascular drainage back to the ILC or MCA in approximately 60% of cases, but in the remaining 40% a more extensive and proximal dissection to reach the SMA itself will be necessary. The surgeon must also acknowledge the occurrence of a second RCA once in every 10–12 operations that he or she performs, thus accepting the potential to leave behind lymphatics with tumour deposits should only one RCA be identified. Preoperative bolus-triggered arterial phase computerised tomography (CT) scanning may in the future provide the necessary vascular detail to identify multiple RCAs. With advancements such as this, minimally invasive precision surgery based on the principles of open CME may become more feasible, as will achieving a residual tumour classification grade 0 (R0 resection), the ‘holy grail’ of oncosurgery [32]. This may lead to better long-term outcomes for patients and emphasises the importance of possessing an intimate knowledge of the underlying vascular anatomy.

It therefore follows that right colon vasculature may be more precisely delineated radiologically, and indeed preoperative vascular mapping is a mainstay of the CME technique. Whilst imaging studies involving the colonic vasculature do generally benefit from larger sample sizes and correlate fairly well with intraoperative data, attempts to corroborate radiological findings anatomically through cadaveric dissection have thus far yielded a statistically significant *incongruence* in results [22]. As an example, the incidence of the MCA and right colic feeding vessel arising from a common trunk differed by a factor of 4.7 between these two methods in a recent comparison paper. Furthermore, this difference was not unidirectional: certain vascular patterns were more commonly identified on CT, whilst others were more seen more often during dissection. However, one major weakness of this study was that CT scans were obtained retrospectively from a database and not performed on the same individuals who were dissected. The first direct comparison of preoperative vascular anatomy (as determined by CT scan) and intraoperative findings was published in 2015 [33]. Among 139 patients only 17 were found to have a right colic feeding vessel at surgery, yielding a sensitivity of 85.7%, a specificity of 95.2% and an overall diagnostic accuracy of 97.1% for identifying the right colic vessel on three-dimensional (3D) CT reconstruction. One of the largest 3D CT-based studies examining the right colic vasculature to date only demonstrated a right colic feeding vessel in 179 of 536 patients (33.4%) [13]. It should therefore be noted that the right colic vessel was much more frequently absent in these radiological studies than the cadaveric studies of the current review and highlights a potential problem in identifying this structure using contemporary imaging. It raises the question of whether our current radiological modalities and ability to interpret their outputs is sufficiently detailed to aid in the planning of precision surgery in the region of the right colon. As discussed above, CT scans using bolus-triggered arterial, venous and delayed phases may therefore hold more promise in the future development of CME.

### Limitations

There are some limitations associated with this review. Anatomical cadaveric research is generally constrained by small sample sizes except for some older landmark studies [12, 26]. Given the wide range of numbers of cadavers in each included study, the authors provided weighted averages in an attempt to adjust for the lower reliability of smaller series. The use of inconsistent anatomical terminology and inadequately detailed data demanded a degree of interpretation of some results. For example, some papers describe the RCA as sharing a common trunk with the ILC or MCA [12, 26], whereas others describe the RCA as being a branch of the ILC or MCA [11, 27]. One study described both arrangements in their series, the distinction appearing to be dependent on how distally the RCA arose along the course of the MCA [10]. These arrangements were amalgamated into a single classification for ease of comparison in this study, in which the RCA was considered to be a branch of the MCA.

## Conclusions

The origin of the right colic feeding vessel is highly variable and arises from the SMA, ILC, root of the MCA and RBMC in a decreasing order of frequency. Knowledge of the precise incidence of the RCA arising from each origin is hampered by inconsistencies in nomenclature and reporting methods in the literature. These present a barrier to the development of CME and the integration of current and future research. A simple classification for describing the anatomy of the RCA has been discussed in this paper, but its success relies upon universal acceptance. Attempts to correlate intraoperative findings with those of CT scanning have yielded some promising results; however, the concordance of this data with the cadaveric literature is poor. Consideration could be given to a bolus-triggered arterial phase CT scan with 3D reconstruction to assist the surgeon in the planning of precision oncosurgery to the right colon in the future.

## Appendix

### Search strategies

PubMed:

(“anatomy and histology”[Subheading] OR (“anatomy”[All Fields] AND “histology”[All Fields]) OR “anatomy and histology”[All Fields] OR “anatomy”[All Fields] OR “anatomy”[MeSH Terms]) AND (right[All Fields] AND (“colic”[MeSH Terms] OR “colic”[All Fields]) AND (“arteries”[MeSH Terms] OR “arteries”[All Fields] OR “artery”[All Fields])) 145 results.

Medline:

1. Anatomy.ti,ab; 86,249 results.
2. (right AND colic AND artery).ti,ab; 176 results.
3. 1 AND 2; 30 results.

Embase via Ovid:

1. Anatomy.af (40,008).
2. Right colic artery.af (91).
3. 1 and 2 (19).

Cochrane:

Right colic artery (title, abstract, keywords) 3 results.
